# Dihydrolipoamide dehydrogenase deficiency in five siblings with variable phenotypes, including fulminant fatal liver failure despite good engraftment of transplanted liver

**DOI:** 10.1002/jmd2.12444

**Published:** 2024-08-21

**Authors:** Mihaela Mihaljević, Danijela Petković Ramadža, Tamara Žigman, Ivana Rako, Slobodan Galić, Toni Matić, Filip Rubić, Ivana Čulo Čagalj, Davor Mayer, Ante Gojević, Stanko Ćavar, Marijana Ćorić, Melanie T. Achleitner, Johannes A. Mayr, Ksenija Fumić, Jurica Vuković, Ivo Barić

**Affiliations:** ^1^ Department of Pediatrics University Hospital Center Zagreb Zagreb Croatia; ^2^ Department of Pediatrics University Hospital Center Zagreb and School of Medicine, University of Zagreb Zagreb Croatia; ^3^ Department of Laboratory Diagnostics University Hospital Center Zagreb Zagreb Croatia; ^4^ Department of Pediatrics University Hospital Centre Split and School of Medicine, University of Split Split Croatia; ^5^ Institute of Forensic Medicine and Criminalistics, School of Medicine, University of Zagreb Zagreb Croatia; ^6^ Department of Surgery University Hospital Center Zagreb Zagreb Croatia; ^7^ Department for Pathology and Cytology University Hospital Center Zagreb and School of Medicine, University of Zagreb Zagreb Croatia; ^8^ University Children's Hospital, Paracelsus Medical University Salzburg Austria

**Keywords:** dihydrolipoamide dehydrogenase deficiency, fulminant liver failure, liver transplant, riboflavin responsiveness

## Abstract

Dihydrolipoamide dehydrogenase (DLD) deficiency can, in one of its forms, be a rare cause of acute liver failure. Clinical presentation is nonspecific. Biochemical findings can reflect metabolic block, but vary depending on patient and his condition. Consensus treatment guidelines do not exist. We present a family with five members suffering from DLD deficiency. Patient 1 presented with emesis, mental deterioration, and fulminant hepatic failure, which required high‐urgency liver transplantation. His younger brother, patient 2, experienced unexplained hypoglycemia and metabolic acidosis on the second day after cardiac surgery. Three affected younger siblings were asymptomatic. In patients with acute liver failure of unknown etiology urgent metabolic work‐up should be done, and whole exome sequencing considered. Liver transplantation remains life‐saving treatment option, but its outcome may be dependent on etiology‐specific supportive treatment.

**Synopsis**

This is the first described pediatric patient who underwent liver transplantation due to dihydrolipoamide dehydrogenase deficiency; good liver transplant engraftment may not guarantee patient's recovery in this disease, at least not without etiology specific treatment.

## 1 BACKGROUND

Dihydrolipoamide dehydrogenase (DLD, E3, lipoamide dehydrogenase; EC 1.8.1.4) is a multifunctional mitochondrial matrix enzyme. It is the flavoprotein component of the three α‐keto acid dehydrogenase multienzyme complexes: pyruvate dehydrogenase complex, α‐ketoglutarate dehydrogenase complex, and branched‐chain α‐keto acid dehydrogenase complex, and also the glycine cleavage system.^1^ DLD deficiency is a rare metabolic disorder caused by recessively inherited mutations in the *DLD* gene.^2^ Both homozygous and compound heterozygous mutations have been described.^3^, ^4^, ^5^, ^6^ Clinical presentation varies: (1) predominantly neurological presentation with usual onset in infancy, (2) hepatic presentation, and (3) myopathic presentation.^4^, ^5^, ^6^, ^7^, ^8^ In early onset DLD deficiency, patients typically present with hypotonia, poor feeding, lethargy, emesis, and lactic acidosis.^4^, ^9^ Untreated infants develop encephalopathy, sometimes in form of Leigh syndrome, and usually do not survive initial metabolic decompensation. Isolated liver involvement usually presents as recurrent liver failure during the episodes of high energy demands (fever, fasting, exhaustion) or after excessive protein and fat meals, starting with nausea and emesis,^3^ which leads to metabolic acidosis, hyperammonemia, hepatomegaly, coagulopathy, and frequently encephalopathy. While in surviving infants with early onset manifestations neurologic impairment usually remains,^9^ two outcomes have been described in hepatic presentation: full recovery from the initial crisis, or liver failure leading to fatal outcome, regardless of presenting age. Myopathic presentation has been associated with muscle cramps, weakness, and elevated creatine kinase as the main features.

As none of disease symptoms are specific, clinical diagnosis of DLD deficiency is impossible. Certain biochemical findings reflecting the metabolic block can point to the diagnosis: elevated concentrations of blood lactate and pyruvate, and plasma leucine, isoleucine, valine, and alloisoleucine. Beside increased excretion of lactate and pyruvate, organic acids frequently show increased excretion of 2‐oxoglutaric acid. However, these parameters may be normal between episodes of metabolic decompensation, or nonspecific, particularly as isolated findings.^10^ Therefore, high clinical awareness and either enzyme assay or gene analysis are necessary to prove the diagnosis.

In this paper, we describe to the best of our knowledge for the first time a patient with fulminant liver failure due to DLD deficiency who died after liver transplant. His case taught us both about necessity to consider this disease in the initial diagnostic work‐up of liver dysfunction and about possible lower efficacy of liver transplantation in this diagnosis. Follow‐up of affected siblings with variable clinical course so far, prompted us to perform literature search in order to propose generally applicable treatment approach.

## 2 CASE PRESENTATION

There are five patients among nine siblings in a family of consanguineous parents of Roma origin.

**Patient 1** was a previously healthy 7‐year‐old boy, who was transferred to our intensive care unit from a local hospital due to the sudden onset of emesis and somnolence. He had fever, abdominal pain, and persistent emesis from the day before, and his condition was worsening. Aside from impaired consciousness, clinical examination showed only hepatomegaly (6 cm below right costal margin). Initial laboratory work‐up (Table 1) showed highly elevated serum aspartate aminotransferase (AST), alanine aminotransferase (ALT), prolonged prothrombin time, hyperammonemia, and lactic acidosis indicating liver failure. Patient's consciousness deteriorated rapidly (Glasgow coma score from 13 to 3), followed by cardiac arrest 8 h after admission. He was resuscitated twice in 20 min interval and developed multi‐organ failure during the following 24 h. Due to severe respiratory distress the patient required mechanical ventilation. He developed paralytic ileus, intestinal bleeding, and acute kidney injury. Chest and abdomen CT scan showed bilateral pleural effusions, enlarged liver with extensive hypovascular zones, diffuse bowel wall edema with mucosal hyperemia, and indirect signs of intestinal bleeding. Laboratory examination revealed neither poisoning nor viral infection. As the patient's condition was worsening in spite of intensive treatment, high‐urgency liver transplantation from a cadaveric donor was done on the third day after admission. Macroscopic and histological analysis of liver showed extensive necrosis, predominantly affecting periportal and somewhat less centrilobular region, and severe canalicular cholestasis. After transplantation, laboratory work‐up indicated good graft function (Table 1). However, renal failure was irreversible, he never tolerated sufficient oral intake, was prone to hypothermia, and had respiratory distress even though he was successfully weaned off mechanical ventilation for a period of time. Brain CT scan on 29th post‐transplantation day showed ventricular system enlargement and bilateral thalamic hypodense lesions with central hyperdensity. Eventually, mixed bacterial and fungal sepsis led to death on 39th post‐transplantation day.

**TABLE 1:** Laboratory results of patient 1 before and after liver transplantation.

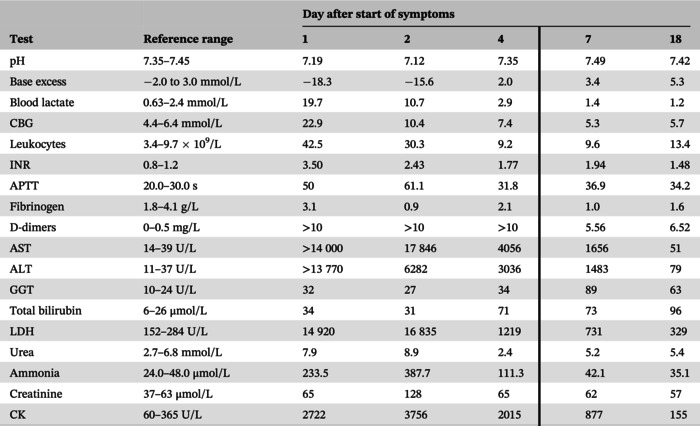	

*Note*: Vertical borderline delineates laboratory results before and after liver transplantation.

Abbreviations: ALT, alanine transaminase; APTT, activated partial thromboplastin time; AST, aspartate aminotransferase; CBG, capillary blood glucose; CK, creatine kinase; GGT, gamma‐glutamyl transferase; INR, international normalized ratio; LDH, lactate dehydrogenase.

**Patient 2** is a younger brother of the first patient. He was born with secundum atrial septal defect (sASD), causing right ventricular and pulmonary artery enlargement, as well as fatigue after mild activity. At the age of 7 he underwent elective surgical closure of sASD. The procedure was uneventful. However, on the second postoperative day he became hypotonic and somnolent due to hypoglycemia (blood glucose 2.5 mmol/L) and had metabolic acidosis (pH 7.28, BE −9.8, HCO_3_ 16.9, lactate 6.4 mmol/L). At that time point the glucose infusion rate was 1.3 mg/kg/min and the patient was nil by mouth, without lipids or amino acids added in the infusion. After intravenous glucose bolus he recovered completely. Laboratory work‐up at the time of hypoglycemia revealed: capillary 3‐OH‐butyrate 0.3 mmol/L, negative urinary ketones, AST 2 × ULN (upper limit of normal), ALT 1.5 × ULN, creatine kinase 3 × ULN, slightly elevated free carnitine in dried blood spot (58.35 mmol/L, ref. interval 10–49.8 mmol/L), plasma free carnitine 2 × ULN, increased excretion of glutaric, malic, fumaric, 2‐hydroxyglutaric, 2‐hydroxyadipic, 2‐oxoadipic, and 2‐oxoglutaric acid in urine. Plasma amino acids showed increased concentrations of: glutamine (1741 μmol/L; ref. interval 254–823), citrulline (91 μmol/L; ref. 1–46), alanine (659 μmol/L; ref. 130–547), glutamic acid (641 μmol/L; ref. 10–150), histidine (130 μmol/L; ref. 41–125), alpha‐aminobutyric acid (63 μmol/L; ref. 4–36), proline (382 μmol/L; ref. 60–340), valine (582 μmol/L; ref. 80–321), isoleucine (171 μmol/L; ref. 22–107), and leucine (423 μmol/L; ref. 49–216). All abnormalities resolved after hypoglycemia was corrected and adequate calorie intake provided. DLD deficiency was diagnosed by whole exome sequencing, and homozygous c.685G>T (p.Gly229Cys) variant was found. Following recovery after cardiac surgery, his exercise tolerance was age‐appropriate, although muscle strength tests revealed mild to moderate muscle weakness. He was discharged with riboflavin supplementation and moderately restricted protein diet. Considering socioeconomic background and level of education of the parents, as well as normal amino acids concentrations under baseline conditions, we decided that optimal treatment would be achieved by recommending reduction of meat, cheese, and eggs intake rather than strict dietary plan. In case of imminent metabolic crisis, we recommended urgent transfer to the nearest hospital, parenteral rehydration with age‐adjusted glucose infusion rate, lipid emulsion 1 g/kg/day, and short‐term discontinuation of protein for up to 48 h, according to Quinonez and Thoene.^11^ As he was stable after initial event, he was followed up in 6 months to 1‐year intervals. At the last evaluation at age of 11 years, he was asymptomatic.

**Patients 3, 4, and 5**, two girls aged 4 and 6, and a 3‐year old boy, respectively, all younger siblings of the first two patients, have been diagnosed with DLD deficiency after family screening for the c.685G>T pathogenic variant. The homozygous mutation was also confirmed post mortem in patient 1. Based on these results, parents received genetic counseling. None of three patients had any symptoms that can be related to DLD deficiency. Laboratory evaluation showed normal complete blood count, coagulation profile, AST, ALT, GGT, ammonia, acid–base balance, albumin, blood lactate, NT‐proBNP, and plasma amino acids. Patients 4 and 5 showed only marginally increased excretion of ethylmalonic acid in urine. Urine organic acid analysis in patient 3 was normal. Patients 3 and 5 had normal abdominal and heart ultrasound, while in patient 4 ultrasound revealed multiseptated gallbladder and slightly spherical left ventricle. Holter electrocardiogram in patient 4 was normal. Riboflavin supplementation and moderate protein restriction diet were recommended for all, as in patient 2, and they were followed up in 6 months to 1‐year intervals. All three were asymptomatic in 2‐year follow‐up period.

### 2.1 Riboflavin responsiveness

**FIGURE 1:**
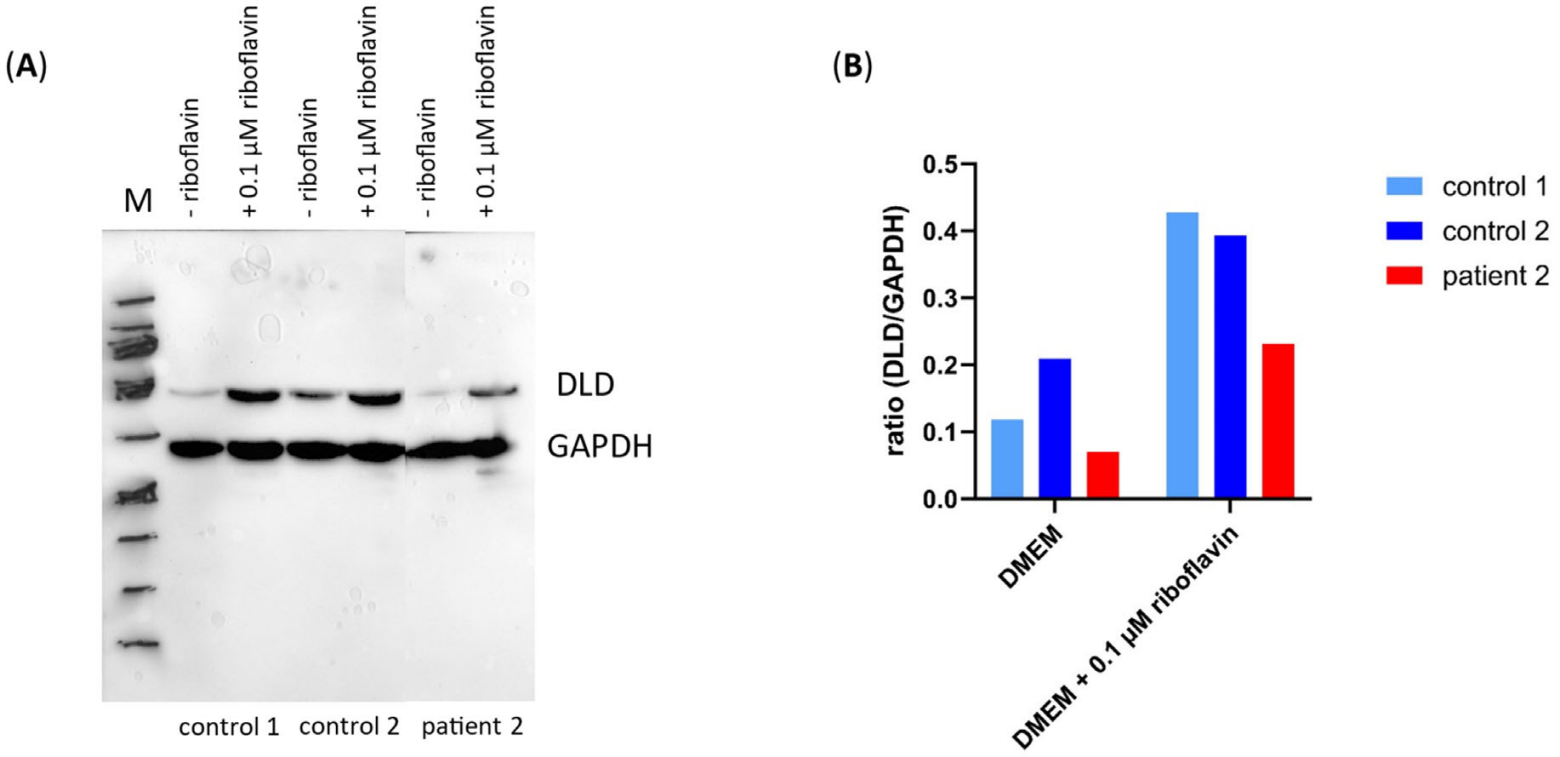
Western blot of whole cell lysates of primary human skin fibroblasts cultured in DMEM or DMEM + 0.1 μM riboflavin. (A) Western blotting of whole cell lysates of two controls and patient 2 harboring DLD deficiency. For loading control GAPDH antibody was used. The blot was exposed for 180 s. (B) Ratio of DLD to GAPDH cells cultured in DMEM were compared to cells cultured in 0.1 μM DMEM + riboflavin. DLD, dihydrolipoamide dehydrogenase; DMEM, Dulbecco's Modified Eagle Medium; GADPH, glyceraldehyde‐3‐phosphate dehydrogenase; M, marker.

Fibroblasts of patient 2 and two controls were grown on DMEM medium and after passaging either shifted to riboflavin‐free DMEM medium or kept on 0.1 μmol/L riboflavin‐containing DMEM medium. After 8 days, the cells were harvested and used for western blotting. As shown in Figure 1, the protein content of DLD was lower in riboflavin‐free medium in both patient and controls. Furthermore, there was a lower amount of DLD in patient cells than in controls.

## 3 DISCUSSION

We report a family with five members suffering from DLD deficiency, which caused metabolic decompensation in two of them, with fatal outcome in one despite high‐urgency liver transplantation with good liver engraftment. This disorder affects primarily tissues with high oxygen consumption, as it is part of three mitochondrial multienzyme complexes (alpha‐ketoglutarate, pyruvate, and branched‐chain alpha‐keto acid dehydrogenase complexes). Even though a loss in DLD function adversely affects multiple key metabolic pathways, severity of clinical presentation rarely reflects residual DLD activity, which implies the involvement of other biochemical mechanisms, and possibly exogenous factors, that could both trigger the symptoms and modify outcomes of this disorder. Enhanced reactive oxygen species generation seems to play roll in pathogenesis as well.^1^

All siblings were homozygous for the c.685G>T pathogenic variant in the *DLD* gene. This mutation has been known to cause DLD deficiency in Ashkenazi Jews with a carrier rate just above 1%,^9^ but was later also found in non‐Ashkenazi patients.^3^, ^12^ The c.685G>T (Gly229Cys) missense variant, located in the NAD+ binding domain of the protein, has been reported in numerous affected individuals with DLD deficiency.^8^, ^12^ The reduced amount and activity of the DLD protein in muscle of homozygotes suggests that the mutation interferes with the stability of the protein.^9^, ^12^ Functional studies showed that the variant increases the generation of reactive oxygen species compared to the wild type enzyme.^13^ Mean residual activity can vary from 7% to 33%, according to Hong et al.^12^ Clinical presentation with prominent hepatic involvement and high survival rate is prevalent in described patients with c.685G>T pathogenic variant.

However, our patient's 1 dramatic onset of fulminant liver and multi‐organ failure resulted in lethal outcome in spite of high‐urgency liver transplantation and critical care treatment, probably reflecting fatal energy breakdown due to DLD deficiency. As his diagnosis was unknown and laboratory results were rather nonspecific, the question is could have earlier diagnosis of DLD deficiency led to more specific treatment approach and possibly different outcome. Unpredictable and rapid onset of symptoms with nonspecific biochemical findings makes it almost impossible to anticipate the diagnosis in previously asymptomatic patient with unremarkable family history. It seems likely that this disease is underdiagnosed in patients with episodic liver dysfunction or failure, suggesting that urgent next generation sequencing, either in the form of whole exome/genome sequencing or acute liver failure gene panel should be included in management algorithms of patients with unexplained acute liver failure, possibly even after transplantation. Similarly, urgent metabolic tests must be included in the work‐up of patients with unexplained liver failure, even if some results may come after emergency liver transplantation, keeping in mind that these results may guide posttransplant treatment and/or help in other family members management or counseling.

Urgent specific treatment during the episodes of metabolic collapse seems to be the only effective method to prevent multi‐organ failure and possible fatal outcome in DLD deficiency. However, the consensus treatment guidelines do not exist. The available recommendations are mostly based on pathogenetic considerations and experiences with small number of patents. Additional challenge is clinical heterogeneity of the disease making universal treatment recommendations hardly applicable. For instance, is it justified to restrict protein intake because of deficiency of the branched‐chain α‐keto acid dehydrogenase complex in patients in whom branched chain amino acids are elevated only mildly and only in crises? Pyruvate dehydrogenase deficiency would normally be treated with ketogenic diet. However, despite the possibility of improving survival, quality of life does not seem to improve.^14^ Furthermore, fat accumulation in liver was reported in some patients with DLD deficiency. Riboflavin supplementation, expecting to have chaperon‐like effect, rarely seemed beneficial. Nevertheless, there is a report of improvement of all signs and symptoms with riboflavin supplementation in a patient with myopathic presentation. Following riboflavin supplementation the reported patient improved physical strength, his muscle biopsy demonstrated the rescue of DLD protein levels and disappearance of histochemical mitochondrial proliferation, increased mitochondria complex V activity, and total celular ATP content.^6^ Unlike our patient with missense variant located in NAD+ binding domain, he was compound heterozygous with one missense mutation located in interface domain of the homodimer, and the other located at the end of mitochondrial targeting sequence and at the proximity of the FAD‐binding domain. Nevertheless, fibroblasts of our patient 2 showed in vitro responsiveness to riboflavin, making riboflavin supplementation in our patients reasonable consideration.

A patient presenting with vomiting associated with neurologic symptoms was reported to have possible beneficial effects following riboflavin treatment. However, the patient received other drugs too, making it unclear if the benefits were from riboflavin.^12^ Treatment suggestions in Gene Reviews recommend the treatment according to main phenotypes and acute versus stable phase of the disease.^11^ However, there are patients with intermediate or changing phenotypes and all possible circumstances influencing the phenotype are unpredictable.

Therefore, so far, it seems that treatment approach should remain individual taking into account severity of mutations, family history, clinical course, biochemical findings, and concurrent circumstances in every single patient. We believe that in hepatic phenotype of DLD deficiency the treatment cornerstone should be prevention/early treatment of metabolic decompensation by avoiding catabolism with sufficient early intravenous glucose intake, if necessary with insulin to avoid lactic acidosis. Intravenous lipids can be added. Energy demands should be reduced. Riboflavin should be supplemented in responsive patients and in possibly responsive ones until responsiveness is not ruled out.

Individual approach is probably recommendable also for liver transplantation in DLD deficient patients, as a procedure which can save life but has in general limited efficacy in mitochondrial energy production disorders. International mulicentric studies and disease‐specific patients registries are highly desirable to help in delineating optimal treatment recommendations.

## AUTHOR CONTRIBUTIONS

Mihaela Mihaljević, Danijela Petković Ramadža, Tamara Žigman, Slobodan Galić, Toni Matić, Filip Rubić, Ivana Čulo Čagalj, Ante Gojević, Stanko Ćavar, Jurica Vuković, and Ivo Barić participated in clinical management of patients. Ivana Rako, Davor Mayer, Marijana Ćorić, and Ksenija Fumić participated in laboratory work‐up of patients. Melanie T. Achleitner and Johannes A. Mayr tested riboflavin‐responsiveness and wrote the related parts of the manuscript. All authors revised and approved the final manuscript as submitted and agree to be responsible for the entire content. Mihaela Mihaljević wrote the manuscript. Ivo Barić designed and led the study.

## FUNDING INFORMATION

This study was supported by the European Joint Programme on Rare Diseases, project EJPRD19‐145 GENOMIT I6478‐B financed by the Austrian Science Fund FWF.

## CONFLICT OF INTEREST STATEMENT

The authors declare no conflicts of interest.

## ETHICS STATEMENT

All procedures followed were in accordance with the ethical standards of the responsible committee on human experimentation (institutional and national) and with the Helsinki Declaration of 1975, as revised in 2000 (5). Informed consent was obtained from parents of patients included in this study.

## Data Availability

The data that support the findings of this study are available from the corresponding author upon reasonable request.
